# Supplementary material for: Associations between self-efficacy, distress and anxiety in cancer patient-relative dyads visiting psychosocial cancer support services: Using actor-partner interdependence modelling
Source: PLoS One. 2021 Sep 17;16(9):e0255318. doi: 10.1371/journal.pone.0255318 (PMC8448356; doi:10.1371/journal.pone.0255318)
Supplement: Attachment — Submitted filename: Response to Reviewers.docx [file pone.0255318.s001.docx]

**Journal Requirements:**

When submitting your revision, we need you to address these additional requirements.

1. Please ensure that your manuscript meets PLOS ONE's style requirements, including those for file naming. The PLOS ONE style templates can be found at 
https://journals.plos.org/plosone/s/file?id=wjVg/PLOSOne_formatting_sample_main_body.pdf and 
https://journals.plos.org/plosone/s/file?id=ba62/PLOSOne_formatting_sample_title_authors_affiliations.pdf

Reply: We have doubled-checked the format of our manuscript and cannot find any deviations from the PLOS ONE style requirements.

2. In your Data Availability statement, you have not specified where the minimal data set underlying the results described in your manuscript can be found. PLOS defines a study's minimal data set as the underlying data used to reach the conclusions drawn in the manuscript and any additional data required to replicate the reported study findings in their entirety. All PLOS journals require that the minimal data set be made fully available. For more information about our data policy, please see http://journals.plos.org/plosone/s/data-availability.

Upon re-submitting your revised manuscript, please upload your study’s minimal underlying data set as either Supporting Information files or to a stable, public repository and include the relevant URLs, DOIs, or accession numbers within your revised cover letter. For a list of acceptable repositories, please see http://journals.plos.org/plosone/s/data-availability#loc-recommended-repositories. Any potentially identifying patient information must be fully anonymized.

Important: If there are ethical or legal restrictions to sharing your data publicly, please explain these restrictions in detail. Please see our guidelines for more information on what we consider unacceptable restrictions to publicly sharing data: http://journals.plos.org/plosone/s/data-availability#loc-unacceptable-data-access-restrictions. Note that it is not acceptable for the authors to be the sole named individuals responsible for ensuring data access.

We will update your Data Availability statement to reflect the information you provide in your cover letter.

Reply: We have made our data available on the public repository OSF. The link to see and download the data is.

<https://osf.io/qfbuc/?view_only=2fa4df7d78d746b9bc41e98a5395440b>

The file shows the original minimal data to replicate our analysis. For an analysis with the APIM_SEM app, it needs to be converted into a dyad format.

Please review your reference list to ensure that it is complete and correct. If you have cited papers that have been retracted, please include the rationale for doing so in the manuscript text, or remove these references and replace them with relevant current references. Any changes to the reference list should be mentioned in the rebuttal letter that accompanies your revised manuscript. If you need to cite a retracted article, indicate the article’s retracted status in the References list and also include a citation and full reference for the retraction notice.

Reply: We have checked every reference. None of the papers we have cited have been retracted.

**Additional Editor Comments:**

Introduction
can you double check the context of the last paragraph and statement that says little is known about... line 116. As a reader I would want to know if you are aware of any work? most importantly that has done something similar to your study, if so name it and identify limitations, if not say to the best of your knowledge none. But also be mindful of other design types which may bring something to the table. Any contextual studies added to give insight to the reader can be taken through to the discussion also. You conclusion says only a few studies have looked at associations line 380 - so please make it clear what is known, not known and what further work is needed and making it easy for the reader to understand your choices.

Reply: Thank you for the remark. We have named a number of articles that have done similar studies in different contexts (situations outside the support context, specific cancer types, male vs female dayds etc.) throughout the introduction and the discussion. However, there are indeed no studies investigating associations between self-efficacy, distress and anxiety in dyads of patients and relatives in the context of seeking psychosocial support. We understand our phrasing in line 116 and 380 may be misleading so we have changed the phrasing in line 116 and deleted the sentence in line 380.

Methods
can you make sure you use sub-heading according to STROBE https://www.strobe-statement.org/index.php?id=available-checklists
for instance take requirements for the area of setting they ask for periods of recruitment, exposure, follow-up, they ask for attempts to address sources of bias, sampling and sample size, also identification of how confounding variables are controlled for in the analysis - you identify gender? wasn't their many others? e.g., mental health, level of physical activity, impact of COVID-19 (sampling period means people could be affected by it) I notice your discussion has some e.g., quality of social support network but I am not sure you cover all possibilities etc. Can you justify your choice and provide limitations if any further are identified as a result of this.

Reply: We have edited the methods section according to the STROBE criteria. The sub-headings *variables*, *data sources/measurement* and *quantitative variables* were grouped together under the sub-heading *instruments*. We have only controlled for gender as our sample size is relatively small and adding more confounders to the analysis would have resulted in a loss of power. Gender was the strongest confounder. We have added this explanation in the limitation section. We have included all participants before the outbreak of COVID-19 in Germany. Furthermore, we believe the mental health of the participants is already sufficiently explained by the variables anxiety and distress. Physical activity was not assessed in the study. Since participants had to be able to travel to the support service facilities, we assume a certain level of physical activity needs to be given. We discuss biases in the limitation section and therefore feel it may be redundant to name them also in the method section. We have added some information on the exposure but did not elaborate on the follow-up since this is a cross-sectional study and may lead to confusion. We have cited the study protocol of the larger study.

**Reviewer #1:**

The authors present a cross-sectional study evaluating self-efficacy, distress and anxiety in dyads of cancer patients and their relatives being advised at psychosocial cancer support centers. This analysis is part of a lager study evaluating efficacy of cancer support centers. The authors were able to analyze a total 42 dyads which is a high number respecting the difficulties to include dyads in prospective studies.
The underlying study concerns on an issue of very high clinical impact as all three factors are frequent and relevant in daily practice in oncologic care of cancer patients and their relatives, but less is known about interaction between this three dimensions and between the two dyad partners.
The manuscript is written interestingly and comprises all relevant previous literature. The study is performed on a high methodological standards and all results are presented in an adequate and comprehensive manner.
In my opinion, this manuscript is suitable for publication in Plos One, but there is one aspect that could improve the overall value of the manuscript and should be revised previously:
The authors should extend their implications for clinical practice. It strengthens the importance of psychosocial cancer support services, but I think there could be more clinical implications. What can oncologist, psycho-oncologist social workers in cancer support services learn from this study for their daily practice? What should they now do to improve their support for cancer patients, their relatives and both. What can they advise them?

Reply: Thank you for reading our manuscript and thank you for the remark. You are right, there are more implications for clinical practice. We have added some more possible implications (see line 349-366).
